# Effects of SGLT‐2 Inhibitors on Plasma Concentrations and Renal Clearance of the Risk Marker Trimethylamine‐N‐Oxide in Patients With Type‐2 Diabetes

**DOI:** 10.1002/edm2.70318

**Published:** 2026-09-30

**Authors:** Katja B. Zimmer, Arne Gessner, Dennis Kannenkeril, Agnes Bosch, Armin Ströbel, Roland E. Schmieder, Renke Maas

**Affiliations:** ^1^ Institute of Experimental and Clinical Pharmacology and Toxicology Friedrich‐Alexander‐Universität Erlangen‐Nürnberg Erlangen Germany; ^2^ FAU NeW – Research Center New Bioactive Compounds Friedrich‐Alexander‐Universität Erlangen‐Nürnberg Erlangen Germany; ^3^ Department of Medicine 4 – Nephrology and Hypertension Universitätsklinikum Erlangen Erlangen Germany; ^4^ Center for Clinical Studies Universitätsklinikum Erlangen Erlangen Germany

**Keywords:** SGLT‐2 inhibitors, trimethylamine N‐oxide, Type 2 diabetes mellitus

## Abstract

**Background:**

Sodium‐glucose cotransporter‐2 (SGLT‐2) inhibitors have become a key drug class in the treatment of Type 2 diabetes (T2D) due to their effectiveness in blood sugar control, but also for many other clinical indications because of beneficial effects on cardiovascular diseases and mortality. Therefore, the findings of a secondary analysis of the recent EMMY trial, in which acute myocardial infarction patients treated with empagliflozin had higher trimethylamine‐N‐oxide (TMAO) serum levels compared to placebo, were unexpected, as high TMAO concentrations in blood have previously been linked to adverse cardiovascular and mortality outcomes. In light of this, the aim of our study was to explore the effects of the SGLT‐2 inhibitors empagliflozin and dapagliflozin on plasma concentrations and renal clearance (Cl_R_) in a T2D patient setting.

**Methods:**

Plasma and 24‐h urine samples from two prospective, randomised, placebo‐controlled, double‐blind, cross‐over trials with T2D patients (empagliflozin *N* = 69, NCT02471963; dapagliflozin *N* = 54, NCT02383238), taken at baseline and after each treatment with either SGLT‐2 inhibitor or placebo, were measured for TMAO via LC–MS/MS.

**Results:**

After treatment with an SGLT‐2 inhibitor, in the total of 123 patients, TMAO plasma levels were 11.5% higher compared to placebo (*p* = 0.006) and Cl_R_ of TMAO was reduced by 10.3% (*p* = 0.048) without any relevant change in its urinary excretion (*p* = 0.521). In the dapagliflozin trial, TMAO plasma concentration was 23.4% higher (*p* = 0.021), and Cl_R_ of TMAO was 22.6% lower (*p* = 0.025) after treatment with dapagliflozin compared to placebo. For empagliflozin, a similar trend was observed, but did not reach statistical significance.

**Conclusion:**

Treatment with an SGLT‐2 inhibitor was associated with a modest increase in plasma concentration of TMAO. Together with data from a previous trial, the present findings suggest a possible class effect of SGLT‐2 inhibitors. Whether this effect impacts the overall clinical benefits of SGLT‐2 inhibitors needs to be subject to further investigations.

**Trial Registration:**

http://www.clinicaltrials.gov: NCT02471963 (registered 15th June 2015, retrospectively registered) and NCT02383238

## Graphical abstract

Treatment with empagliflozin or dapagliflozin was associated with a modest increase in plasma concentration of TMAO in Type 2 diabetes patients. These findings suggest a possible class effect of SGLT‐2 inhibitors. Whether this effect impacts the overall clinical benefits of SGLT‐2 inhibitors needs to be subject to further investigation.

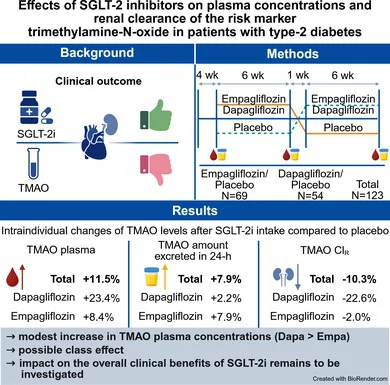

## 1 Background

Sodium‐glucose cotransporter‐2 (SGLT‐2) inhibitors inhibit the reabsorption of glucose from the urine in the proximal renal tubule and thereby lower blood sugar levels by increasing its excretion [1]. Due to their effectiveness, they have become a key drug class in the treatment of Type 2 diabetes (T2D). However, treatment with SGLT‐2 inhibitors, such as empagliflozin and dapagliflozin, showed clinical benefits on cardiovascular outcomes [2, 3, 4, 5, 6] and mortality [7, 8] not only in T2D patients, but also in non‐diabetic patients. For this reason, potential mechanisms beyond glucose control mediating clinical SGLT‐2 effects have come into focus [9, 10].

One potential mechanism could be that SGLT‐2 inhibitors reduce endogenous and potentially harmful substances produced by the gut microbiome. According to a study by Billing et al., the concentration of uremic toxins decreased under therapy with SGLT‐2 inhibitors in mice and in addition, there was a broad downregulation of transport proteins in the kidney [11]. With this in mind, the cardiovascular and renal risk marker trimethylamine‐N‐oxide (TMAO) becomes a subject of interest.

TMAO is a small, low‐weight metabolite, found in foods such as saltwater fish, and generated from trimethylamine (TMA) by oxidation by hepatic flavin monooxygenase 3 (FMO3) [12, 13, 14]. TMA is produced by the gut microbiome from dietary precursors, such as choline, phosphatidylcholine, betaine, and L‐carnitine [12, 13]. Elevated plasma TMAO levels have already been associated with an increased risk of various diseases, including cardiovascular disease, chronic kidney disease, atherosclerosis, and T2D [15, 16, 17, 18]. In T2D patients in particular, increased levels of TMAO have been linked to a greater risk of cardiovascular events, all‐cause mortality, and a decline in kidney function [12, 19, 20].

In a secondary analysis of the EMMY trial exploring underlying effects beyond glucose control, it was found that patients treated with empagliflozin exhibited a more pronounced increase in serum TMAO levels compared to those who received placebo [21].

In view of this observation, the aim of the current study was to investigate whether this effect also occurs in T2D patients and extends to other clinical settings and other widely used SGLT‐2 inhibitors, such as dapagliflozin. Here, two recent placebo‐controlled trials (NCT02471963 and NCT02383238), which assessed vascular effects of SGLT‐2 inhibitors as well as the renal clearance (Cl_R_) in T2D patients, offered the opportunity to evaluate their effect on TMAO plasma concentration as well as TMAO Cl_R_.

## 2 Methods

### 2.1 Study Design

For this study, individual patient data and samples of two prospective, randomised, placebo‐controlled, double‐blind cross‐over trials of empagliflozin and dapagliflozin in patients with T2D were combined (NCT02471963 and NCT02383238). The exact details of the design, methods and patient characteristics of these two studies with comparable populations have already been published [22, 23, 24, 25, 26] an overview is provided in the supplemental file. To provide a concise overview: In both trials, patients were randomised after a run‐in/washout phase to receive either 25 mg empagliflozin/10 mg dapagliflozin or placebo orally once daily for six weeks. This was followed by a washout phase of one week before patients switched to receive either placebo or active drug for another six weeks. The placebos used in the clinical trials are matching/identical film‐coated tablets that contain the same inactive ingredients as the active tablets but omit the active substance empagliflozin and dapagliflozin. The tablets are designed to be identical in appearance, size, shape, colour, and taste to maintain blinding. Tablets with active drug and placebo were produced and delivered to our clinical research centre by the pharmaceutical companies. Details on the composition can be found in the official product information [27, 28] and in the additional file. Collection of fasting blood and 24‐h urine samples took place at baseline and at the end of both six‐week periods. No preservatives were used in the urine collection containers. All samples were stored at −80°C and thawed directly before analysis. Also, all concomitant therapies were recorded throughout the study. The local ethics committee (University of Erlangen‐Nuremberg, numbers 360_14 Az and 277_14 Az) and the German drug authority approved the study protocols, and the study was conducted in accordance with the Declaration of Helsinki and the principles of good clinical practice guidelines. All participants provided written informed consent.

### 2.2 Analysis of Plasma and Urine Samples

Laboratory measurements, including serum creatinine, were performed by the accredited central laboratory of the University Hospital Erlangen. The central laboratory uses the Photometric Jaffe method for serum creatinine measurements.

The quantification of TMAO was performed at the Institute of Experimental and Clinical Pharmacology and Toxicology (Friedrich‐Alexander‐Universität Erlangen‐Nürnberg, Erlangen, Germany) as follows: Of the 71 participants in the empagliflozin trial, plasma and 24‐h urine samples were available from 70 participants, and of the 59 participants in the dapagliflozin trial, plasma and 24‐h urine samples were available from 56 participants at the time points analysed. The TMAO concentration was assessed for all samples using a previously published and validated LC–MS/MS assay [29]. In brief, 20 μL of a plasma sample was precipitated with 100 μL of methanol containing 0.25 μmol/L TMAO‐d9 as an internal standard. Following centrifugation (3 min, 5°C, 24,940 g), 100 μL of the resulting supernatant was diluted with 200 μL acetonitrile and subsequently analysed. For urine samples, 20 μL were first diluted with 400 μL urine imitate (Sigmatrix Urine Diluent, Sigma‐Aldrich/Merck, Darmstadt, Germany) containing 8 μmol/L internal standard. Then, according to the processing of the plasma samples, 20 μL of this dilution was precipitated with 100 μL methanol, centrifuged and 100 μL of supernatant was mixed with 400 μL acetonitrile for quantification. As the TMAO concentration could not be quantified in one urine sample in the empagliflozin trial and in two urine samples in the dapagliflozin trial, these individuals were excluded from the final study population and downstream data analysis. Accordingly, the final analytical sample comprised 69 patients from the empagliflozin trial and 54 patients from the dapagliflozin trial, resulting in a total of 123 patients.

### 2.3 Statistics and Calculations

Statistical analysis was performed using IBM SPSS Version 30. The figures were created using GraphPad Prism 5. A *p*‐value of < 0.05 (two‐sided) indicated statistical significance. Compliance was measured as follows: (Number of tablets actually taken/Number of tablets to be taken)*100. The estimated glomerular filtration rate (eGFR) was calculated based on creatinine concentrations using the CKD‐EPI 2021 equation [30]. The following formula was used to calculate Cl_R_, taking into account the determined concentrations in plasma and urine and the volume of 24‐h urine:$\;\left(c_{\text{urine}}\times V_{\text{urine}}\right)/\left(c_{\text{plasma}}\times 1440\;\min\right)$. Crossover‐difference was calculated by subtracting TMAO concentrations after administration of placebo from TMAO concentration after treatment with an SGLT‐2 inhibitor. Intraindividual percentage change of active drug treatment compared to placebo was calculated as follows: (Crossover‐difference/TMAO value after Placebo)*100%.

Since TMAO concentrations were not normally distributed, as assessed by histograms and normal probability plots, two‐sided Wilcoxon signed‐rank tests were performed to compare absolute TMAO levels after placebo and active drug treatment. One‐sample Wilcoxon signed‐rank test with a hypothesised median value of zero was performed to calculate *p*‐values of intraindividual percentage change. Mann–Whitney U test was used to evaluate differences in crossover differences between the subgroups of patients with and without intake of an antibiotic during the trial. Two‐sided paired *t*‐tests were used for normally distributed parameters.

In an exploratory analysis, Spearman‐Rho correlations were calculated between TMAO plasma concentration or Cl_R_ and eGFR and fasting blood glucose (FPG) at baseline, after treatment with placebo and after treatment with empagliflozin or dapagliflozin. Furthermore, correlations with the intraindividual crossover difference in plasma concentration or Cl_R_ on the one hand and intraindividual crossover differences of eGFR and FPG on the other hand were assessed.

## 3 Results

### 3.1 Patients

The detailed patient characteristics have already been published for both underlying trials [22, 23, 24, 25]. In brief, the mean age of the 123 patients from both trials included in this analysis was 61.2 years, and 59% of the participants were male. Furthermore, the mean baseline glycated haemoglobin (HbA1c) was 6.7%, and the mean eGFR was 93.8 mL/min/1.73m^2^, being above 90 mL/min/1.73m^2^ for 70.7% of the patients (Table 1). In both trials, the mean compliance during intervention was 99%, with a minimum of 87%.

**TABLE 1:** Baseline characteristics.

Variables	Dapagliflozin trial (*N* = 54)	Empagliflozin trial (*N* = 69)	Total (*N* = 123)
Age (years), mean ± SD	60.4 ± 7.8	61.8 ± 6.8	61.2 ± 7.3
Sex, *n* (%)			
Male	32 (59.3)	41 (59.4)	73 (59.3)
Female	22 (40.7)	28 (40.6)	50 (40.7)
Body mass index (kg/m^2^), mean ± SD	29.6 ± 4.3	30.2 ± 4.2	29.9 ± 4.2
HbA1c (%), mean ± SD	6.6 ± 0.7	6.7 ± 0.8	6.7 ± 0.8
Fasting blood glucose (mg/dL)	129.7 ± 26.9	139.4 ± 31.0	135.2 ± 29.5
eGFR (mL/min/1.73m^2^), mean ± SD	93.7 ± 12.0	93.8 ± 10.9	93.8 ± 11.3
eGFR categories, *n* (%)			
< 60	0 (0)	0 (0)	0 (0)
60–90	16 (29.6)	20 (29.0)	36 (29.3)
> 90	38 (70.4)	49 (71.0)	87 (70.7)

Abbreviations: HbA1c, glycated haemoglobin; SD, standard deviation.

### 3.2 Changes in eGFR

The changes in eGFR are shown in Table 2. In summary, a decrease in eGFR after treatment with an SGLT‐2 inhibitor compared to placebo was observed, which was statistically significant for the total of 123 patients (*p* < 0.001) and for the group of 69 patients receiving empagliflozin (*p* < 0.001), but not for the 54 participants receiving dapagliflozin (*p* = 0.058).

**TABLE 2:** TMAO data in plasma and urine and renal clearance of TMAO after empagliflozin/dapagliflozin.

	At baseline	After placebo	After intervention	Crossover difference	*p*
Total (*N* = 123)					
Plasma TMAO (μmol/L)	3.71 (2.57–5.71)	3.60 (2.56–5.20)	4.19 (2.91–6.17)	0.36 (−0.56–1.76)	0.006
Amount excreted in 24 h urine (μmol)	537.2 (359.0–1005.3)	518.6 (343.9–780.0)	537.6 (363.6–776.8)	24.3 (−184.6–196.7)	0.521
Renal Clearance (mL/min)	106.4 (73.6–155.1)	95.8 (74.3–148.4)	83.9 (61.8–120.5)	−9.5 (−49.9–24.0)	0.048
eGFR (mL/min/1.73m^2^)	93.8 ± 11.3	93.2 ± 11.6	90.5 ± 13.0	−2.6 ± 6.4	< 0.001
24 h urine volume (L)	2.2 ± 0.8	2.3 ± 0.8	2.5 ± 0.9	0.2 ± 0.8	0.005
Empagliflozin (*N* = 69)					
Plasma TMAO (μmol/L)	4.08 (2.58–5.95)	3.80 (2.78–5.48)	4.00 (2.90–6.19)	0.22 (−0.72–1.56)	0.102
Amount excreted in 24 h urine (μmol)	505.4 (342.1–1000.5)	490.9 (339.2–670.3)	519.2 (328.8–722.1)	24.3 (−175.4–190.7)	0.536
Renal Clearance (mL/min)	97.2 (64.9–129.1)	87.7 (63.9–123.1)	79.5 (56.3–120.5)	−1.9 (−29.1–33.4)	0.528
eGFR (mL/min/1.73m^2^)	93.8 ± 10.9	93.1 ± 10.9	89.8 ± 12.9	−3.3 ± 6.0	< 0.001
24 h urine volume (l)	2.2 ± 0.9	2.3 ± 0.8	2.5 ± 0.8	0.1 ± 0.8	0.142
Dapagliflozin (*N* = 54)					
Plasma TMAO (μmol/L)	3.41 (2.49–5.41)	3.30 (2.39–5.12)	4.44 (2.91–5.91)	0.58 (−0.54–2.10)	0.021
Amount excreted in 24 h urine (μmol)	570.5 (384.2–1008.1)	540.9 (344.9–862.0)	581.8 (400.7–784.5)	0.4 (−275.8–219.4)	0.734
Renal Clearance (mL/min)	117.8 (76.7–165.9)	109.2 (85.4–164.9)	97.8 (66.4–123.9)	−22.1 (−55.3–17.0)	0.025
eGFR (mL/min/1.73m^2^)	93.7 ± 12.0	93.3 ± 12.5	91.5 ± 13.2	−1.8 ± 6.9	0.058
24 h urine volume (L)	2.1 ± 0.8	2.2 ± 1.0	2.5 ± 0.9	0.3 ± 0.8	0.012

*Note:* Data are median (IQR) for eGFR and 24 h urine volume; mean ± SD; *p*‐values calculated by Wilcoxon signed‐rank test for eGFR and 24 h urine volume by two‐sided paired *t*‐test; *p*‐values refer to absolute changes between placebo and intervention. eGFR was calculated using the CKD‐EPI (2021) formula.

Abbreviations: IQR, interquartile range; SD, standard deviation; TMAO, trimethylamine‐N‐oxide.

### 3.3 Plasma Concentrations of TMAO

As summarised in Table 2, the median TMAO plasma concentration for the total of 123 patients was 3.71 (Q1–Q3: 2.57–5.71) μmol/L at baseline and 3.60 (Q1–Q3: 2.56–5.20) μmol/L after treatment with placebo as compared to 4.19 (Q1–Q3: 2.91–6.17) μmol/L (*p* = 0.006) after treatment with an SGLT‐2 inhibitor. The median intraindividual percentage change was 11.5 (Q1–Q3: −14.1–60.6) % with a *p*‐value of *p* < 0.001, indicating that the observed median differs significantly from the hypothetical median of zero (Figure 1). For the 69 participants of the empagliflozin trial, the median TMAO plasma concentration was 3.80 (Q1–Q3: 2.78–5.48) μmol/L after treatment with placebo and increased to 4.00 (Q1–Q3: 2.90–6.19) μmol/L after receiving empagliflozin (*p* = 0.102) with a median intraindividual percentage change of 8.4 (Q1–Q3: −24.4–51.0) % (*p* = 0.021). In the dapagliflozin trial with 54 patients, the median TMAO plasma concentration after receiving placebo was 3.30 (Q1–Q3: 2.39–5.12) μmol/L and increased significantly to 4.44 (Q1–Q3: 2.91–5.91) μmol/L (*p* = 0.021) with a median intraindividual percentage change of 23.4 (Q1–Q3: −12.3–75.1) % (*p* = 0.002) after receiving dapagliflozin.

**FIGURE 1:**
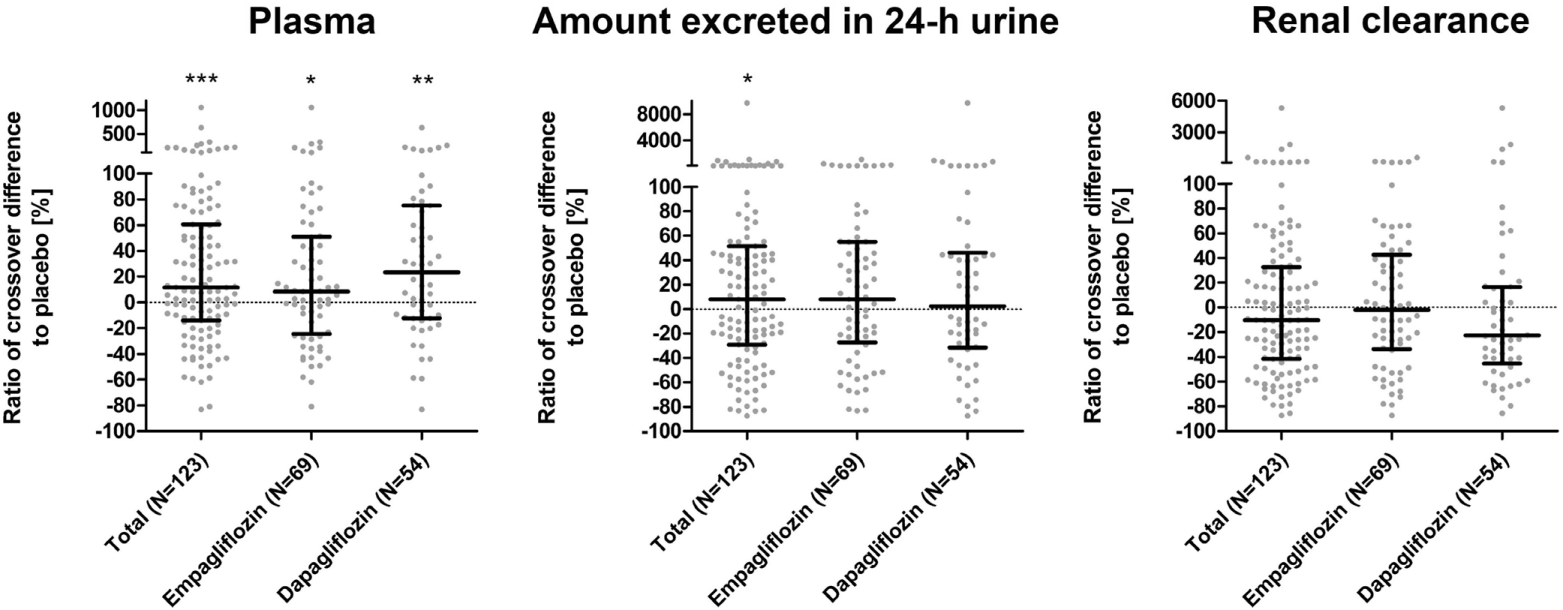
Intraindividual percentage ratios of crossover differences in TMAO values after treatment with an SGLT‐2 inhibitor in comparison to placebo values. *p*‐values calculated by a one‐sample Wilcoxon signed‐rank test with a hypothesised median value of zero. *p < 0.05; **p < 0.01; ***p < 0.001.

### 3.4 Renal Excretion and Renal Clearance of TMAO

The median amount of TMAO excreted in the 24‐h urine for the total of 123 participants was 518.6 (Q1–Q3: 343.9–780.0) μmol after the administration of placebo. After treatment with an SGLT‐2 inhibitor the amount of TMAO excreted in the 24‐h urine increased numerically with a median intraindividual percentage change of 7.9 (Q1–Q3: −29.2–51.3) % (*p* = 0.047, Figure 1) compared to placebo, but the absolute increase of TMAO excretion did not reach statistical significance (24.3 (Q1–Q3: −184.6–196.7) μmol *p* = 0.521). Among the 69 patients receiving empagliflozin the median amount of TMAO excreted in 24‐h urine after treatment with placebo was 490.9 (Q1–Q3: 339.2–670.3) μmol and an increase of TMAO excreted after treatment with empagliflozin to 519.2 (Q1–Q3: 328.8–722.1) μmol (*p* = 0.536), with a median intraindividual percentage change of 7.9 (Q1–Q3: −27.4–54.9) % (*p* = 0.111) compared to placebo, was observed. In the dapagliflozin trial the median amount of TMAO excreted in the 24‐h urine was 540.9 (Q1–Q3: 344.9–862.0) μmol after application of placebo and 581.8 (Q1–Q3: 400.7–784.5) μmol after administration of dapagliflozin (*p* = 0.734), this corresponds to an increase of the amount of TMAO excreted in 24‐h urine with an intraindividual percentage change of 2.2 (Q1–Q3: −31.6–46.2) % (*p* = 0.220). For the total of 123 patients, the median Cl_R_ of TMAO was 95.8 (Q1–Q3: 74.3–148.4) mL/min after receiving placebo. After treatment with an SGLT‐2 inhibitor, the median Cl_R_ of TMAO declined to 83.9 (Q1–Q3: 61.8–120.5) mL/min (*p* = 0.048) with a median intraindividual percentage change of −10.3 (Q1–Q3: −41.7–32.6) % (*p* = 0.376). In the empagliflozin trial the median Cl_R_ of TMAO was 87.7 (Q1–Q3: 63.9–123.1) mL/min after administration of placebo and decreased to 79.5 (Q1–Q3: 56.3–120.5) mL/min after treatment with empagliflozin (*p* = 0.528), with a median intraindividual percentage change of −2.0 (Q1–Q3: −33.7–42.6) % (*p* = 0.827). Among the 54 participants of the dapagliflozin trial the median Cl_R_ of TMAO decreased significantly from 109.20 (Q1–Q3: 85.4–164.9) mL/min after receiving placebo to 97.8 (Q1–Q3: 66.4–123.9) mL/min after administration of dapagliflozin (*p* = 0.025), with a median intraindividual percentage change of −22.6 (Q1–Q3: −45.4–16.6) % (*p* = 0.106).

### 3.5 Correlation of Treatment‐Related Changes

On an exploratory basis, we assessed correlations between TMAO plasma concentrations and eGFR and FPG, as well as TMAO Cl_R_ and eGFR and FPG. We found negative correlations between TMAO plasma values and eGFR at all three time points and no correlation with FPG (additional file Table S1 to Table S3). We also found correlations between Cl_R_ of TMAO and eGFR at all three time points and no correlation with FPG (additional file Table S4 to Table S6). A further analysis between interindividual crossover differences of TMAO plasma concentration or Cl_R_ on one hand and intraindividual crossover differences of eGFR and FPG on the other hand showed no significant correlation (additional file Table S7 and Table S8).

### 3.6 Impact of Concomitant Intake of Antibiotics

As the gut microbiome may affect TMAO, we assessed whether taking an antibiotic influenced the results by conducting a subgroup analysis in which participants who had taken an antibiotic at least once at any time point during the study (9 participants in the dapagliflozin trial and 11 participants in the empagliflozin trial) were excluded from the analysis. There was no statistically significant difference in crossover differences of TMAO plasma values, amount of TMAO excreted in 24‐h urine, and Cl_R_ between the total of participants and those with intake of an antibiotic and also not between participants with no intake of an antibiotic and participants with intake of an antibiotic (additional file Table S9 and Table S10).

## 4 Discussion

In the present study, we evaluated the impact of the SGLT‐2 inhibitors empagliflozin and dapagliflozin on plasma concentration and Cl_R_ of TMAO in a T2D patient setting. In plasma, TMAO levels were slightly and statistically significantly higher, and Cl_R_ of TMAO was modest but statistically significantly reduced in the total population receiving either empagliflozin or dapagliflozin and in the subgroup population receiving dapagliflozin. In the subgroup treated with empagliflozin, a numerically similar trend was observed but did not reach statistical significance. A secondary analysis of the EMMY trial also observed a higher increase in serum TMAO levels in post‐myocardial infarction patients treated with empagliflozin compared to placebo [21]. The present data are in line with this observation, even though statistical significance was not achieved in the empagliflozin subgroup, which could be due, among other things, to lower patient numbers and different health conditions at baseline compared to the EMMY trial. The present study extends this finding to treatment with dapagliflozin, which apparently led to an even greater increase in TMAO levels than that observed with treatment with empagliflozin within 6 weeks of active drug treatment. Taken together, these findings suggest a possible class effect of SGLT‐2 inhibitors.

A potential mechanism for this effect could be a treatment‐induced decline in Cl_R_ of TMAO. The Cl_R_ of TMAO observed in our trial is lower than 218 mL/min reported by Hai et al. [31] but is consistent with the findings of other studies conducted by Miyake et al. [32] and Pelletier et al. [33]. TMAO is eliminated via the urine without undergoing any relevant biotransformation [13, 34]. The predominant route of renal excretion is believed to be glomerular filtration with a considerable contribution of transporter‐mediated secretion, and elevated TMAO plasma concentrations have been previously linked to decreased kidney function [18, 31, 32, 33, 35, 36, 37].

Our analysis also found an association between lower eGFR and higher TMAO plasma concentrations, as well as lower TMAO Cl_R_. Accordingly, a reduction in eGFR could explain the decreased Cl_R_ of TMAO and elevated TMAO plasma concentrations. Moreover, SGLT‐2 inhibitors have been known to induce a reversible dip in eGFR shortly after initiation [27, 28, 38, 39]. However, while a slight dip in eGFR was observed in the present study, the intraindividual reduction in eGFR was marginal in the dapagliflozin group in particular, which exhibited more pronounced changes in TMAO plasma concentration and Cl_R_ than the empagliflozin group. In addition, no correlation was found between intraindividual crossover differences in eGFR and TMAO plasma concentration or Cl_R_. Therefore, a reduction in eGFR may not provide a sufficient explanation for the changes in TMAO levels after administration of an SGLT‐2 inhibitor. Other studies suggest that TMAO is also eliminated by renal secretion, and organic cation transporter 2 (OCT2) and multidrug and toxin extrusion protein 1 (MATE1) have been identified to be involved in transporter‐mediated excretion of TMAO [31, 32, 36, 37]. Both transport proteins are expressed in renal proximal tubule cells, where OCT2 is located in the basolateral membrane and mediates the uptake of TMAO from the blood into the cell, whereas MATE1 is located in the apical membrane, mediating the transport of TMAO out of the cell into the urine [32, 36, 37, 40, 41]. While it is well known that transport proteins play a vital role in the distribution and elimination of both endogenous and exogenous substances, it is also established that exogenous substances, including some widely used drugs, can influence the function of transport proteins [42, 43]. Therefore, the possibility of an interaction between empagliflozin or dapagliflozin and OCT2‐ or MATE1‐mediated transport must be considered, as this could explain the increased plasma concentrations and reduced Cl_R_ of TMAO observed. Also, it was shown that treatment with SGLT‐2 inhibitors led to a downregulating of many transporters and caused a broad functional remodelling in the proximal tubules [11]. The data available on whether empagliflozin or dapagliflozin interact with OCT2 and/or MATE1 is rather limited. The official product information provided by the European Medicines Agency states that empagliflozin is not a substrate of OCT2 [28]. Any inhibition regarding empagliflozin in relation to MATE1 and dapagliflozin in relation to both transporters is not addressed [27, 28]. However, since the amount of TMAO excreted within 24‐h via the urine was barely affected by SGLT‐2 treatment, alterations in transport protein‐mediated excretion (or uptake) do not seem to provide a sufficient explanation for the increased TMAO plasma concentrations.

Therefore, additional factors, such as diet and the composition of the gut microbiome, that are known to influence TMAO concentrations, have to be taken into consideration [12]. Taking antibiotics can disrupt the gut microbiome [44]; however, the subgroup analysis revealed no significant difference in the TMAO crossover differences between participants who had taken an antibiotic and those who had not. Besides that, consumption of a diet rich in TMAO and its precursors, such as animal products like red meat, eggs, dairy products, and saltwater fish, is associated with increased TMAO plasma concentrations [12, 14, 29, 45]. Here, the crossover design of both underlying studies, with participants serving as their own control groups, makes diet an unlikely confounding factor. Some studies demonstrated that treatment with SGLT‐2 inhibitors can influence the composition of the gut microbiome and thus lead to a reduction in bacteria involved in the production of various metabolites, including TMAO [11, 21, 46, 47]. Hence, it was presumed that TMAO levels would decrease or remain stable with SGLT‐2 inhibitor administration, yet a study by Aziz et al. [21] and the present study indicate that this is not the case.

TMAO has been previously characterised in numerous studies as a risk marker and putative risk factor for a variety of diseases, including cardiovascular disease and chronic kidney disease [12]. The role of TMAO in the context of T2D has been the subject of considerable research efforts as well, with a growing body of evidence suggesting a correlation between TMAO levels and the risk of developing T2D, as well as an association of elevated TMAO concentrations and an increased risk of cardiovascular events, a decline in renal function and increased all‐cause mortality [12, 19, 20, 48, 49, 50]. In this context, further studies are needed to investigate, in particular, whether the effect of SGLT‐2 inhibitors on TMAO also modulates the effect of SGLT‐2 inhibitors on clinical outcomes. Here, exploring the effect on outcome or benefit conveyed by SGLT‐2 inhibitors in relation to baseline TMAO may provide further insights. Furthermore, it would be of interest to investigate whether this phenomenon also occurs in patients with much higher baseline TMAO levels, or if the effect of SGLT‐2 inhibitors differs in such cohorts.

## 5 Limitations

The present study has limitations that need to be considered when interpreting its results: Firstly, the observation period of six weeks per treatment is rather brief. Consequently, it is currently not possible to draw definitive conclusions about long‐term effects, including whether plasma TMAO levels will continue to increase, whether the effect will be limited at some point, or whether it may decline again. In addition, a longer treatment period could be beneficial to adjust for potential confounders such as the well‐known initial drop in eGFR [27, 28, 38, 39]. Moreover, following the initial treatment phase and the subsequent washout period, no second baseline was established at the onset of the second treatment phase in both underlying trials to minimise patient burden. Consequently, potential carryover effects persisting significantly longer than the direct drug effect may have attenuated effects in patients randomised to the treatment order active drug first, placebo second. Also, while all concomitant medications were recorded during the trials, the participants' diets were not documented. Therefore, possible effects of diets on TMAO levels cannot be investigated in the current study. In addition, larger sample sizes are required to account for this aspect. A further limitation is that compliance was assessed by enumerating the tablets, which leaves the possibility that patients discarded them instead of taking them. Finally, patients with an eGFR < 60 mL/min/1.73m^2^ were excluded from participating in either of the two trials [22, 23, 24, 25]. Therefore, no conclusion can be drawn regarding the impact of administering SGLT‐2 inhibitors on TMAO values in patients with impaired renal function.

## 6 Conclusion

This pooled analysis showed that treatment with empagliflozin or dapagliflozin was associated with modest increases in TMAO plasma concentration. Combined with observations from the EMMY trial, these data suggest a possible class effect [21]. The most plausible mechanism for this effect appears to be an increased TMAO production, as the amount excreted in 24‐h urine remained largely unchanged. Further studies are, however, needed to confirm this hypothesis and to assess possible interactions of the effects on TMAO and the overall clinical benefits of SGLT‐2 inhibitors.

## Author Contributions

**Armin Ströbel:** formal analysis, writing – review and editing. **Arne Gessner:** investigation, methodology, writing – review and editing. **Agnes Bosch:** writing – review and editing, resources. **Renke Maas:** conceptualization, supervision, formal analysis, resources, writing – review and editing. **Dennis Kannenkeril:** writing – review and editing. **Roland E. Schmieder:** writing – review and editing. **Katja B. Zimmer:** investigation, formal analysis, writing – original draft, writing – review and editing.

## Funding

The DAPA trial (NCT02383238) was an investigator‐initiated clinical trial, with funding provided by AstraZeneca to the University Hospital, Erlangen, Germany. The EMPA trial (NCT02471963) was an investigator initiated clinical trial supported by a grant provided by Boehringer Ingelheim International GmbH.

## Ethics Statement

The study protocol of each trial was approved by the German drug authority and the Local Ethics Committee (University of Erlangen‐Nürnberg), and the studies were conducted in accordance with the Declaration of Helsinki and the principles of Good Clinical Practice guidelines.

## Consent

Written informed consent was obtained from each patient before study inclusion.

## Conflicts of Interest

The authors declare no conflicts of interest.

## Supporting information


**Table S1:** Intraindividual correlations of plasma TMAO values at baseline.
**Table S2:** Intraindividual correlations of plasma TMAO values after treatment with an SGLT2‐inhibitor.
**Table S3:** Intraindividual correlations of plasma TMAO values after treatment with placebo.
**Table S4:** Intraindividual correlations of renal clearance of TMAO at baseline.
**Table S5:** Intraindividual correlations of renal clearance of TMAO after treatment with an SGLT2‐inhibitor.
**Table S6:** Intraindividual correlations of renal clearance of TMAO after treatment with placebo.
**Table S7:** Intraindividual correlations of treatment‐related change in plasma after treatment with an SGLT2‐inhibitor in comparison to treatment with placebo.
**Table S8:** Intraindividual correlations of treatment‐related change in renal clearance after treatment with an SGLT2‐inhibitor in comparison to treatment with placebo.
**Table S9:** Influence of antibiotics—comparison between total participants and participants with no intake of an antibiotic.
**Table S10:** Influence of antibiotics—comparison between participants with no intake of an antibiotic and participants with intake of an antibiotic.

## Data Availability

The data that support the findings of this study are available on request from the corresponding author. The data are not publicly available due to privacy or ethical restrictions.
