# Supplementary material for: Effects of SGLT‐2 Inhibitors on Plasma Concentrations and Renal Clearance of the Risk Marker Trimethylamine‐N‐Oxide in Patients With Type‐2 Diabetes
Source: Endocrinol Diabetes Metab. 2026 Sep 30;9(6):e70318. doi: 10.1002/edm2.70318 (PMC13627872; doi:10.1002/edm2.70318)
Supplement: Supplementary file 1 [file EDM2-9-e70318-s001.docx]

**Supplement Information**

**Effects of SGLT-2 inhibitors on plasma concentrations and renal clearance of the risk marker trimethylamine-N-oxide in patients with type-2 diabetes**

Katja B. Zimmer^1^, Arne Gessner^1,2^ Dennis Kannenkeril^3^, Agnes Bosch^3^, Armin Ströbel^4^, Roland E. Schmieder^3^, Renke Maas^1,2^

^1^ Institute of Experimental and Clinical Pharmacology and Toxicology, Friedrich-Alexander-Universität Erlangen-Nürnberg, Erlangen, Germany

^2^ FAU NeW – Research Center New Bioactive Compounds, Friedrich-Alexander-Universität Erlangen-Nürnberg, Erlangen, Germany
^3^ Department of Medicine 4 – Nephrology and Hypertension, Universitätsklinikum Erlangen, Erlangen, Germany

^4^ Center for Clinical Studies, Universitätsklinikum Erlangen, Erlangen, Germany

Address for correspondence: Prof. Dr. Renke Maas, Institute of Experimental and Clinical Pharmacology and Toxicology, Friedrich-Alexander-Universität Erlangen-Nürnberg, Fahrstr. 17, 91054 Erlangen, Germany. Tel: +49 9131 85 22754; E-Mail: renke.maas@fau.de

**Further information on study design**^1–5^

|  | Dapagliflozin trial | Empagliflozin trial |
| --- | --- | --- |
| Inclusion criteria | - Type 2 diabetes mellitus defined by fasting glucose ≥126 mg/dl or HbA1c ≥6.5% or on blood glucose lowering medication - Age of 18 - 75 years - Male and Female patients are eligible. Females of child bearing potential (WOCBP)1 are only eligible if pregnancy test at the screening visit is negative and they use adequate contraceptive precautions during the trial. | - Type 2 diabetes mellitus defined by fasting glucose ≥ 126 mg/dl or HbA1c ≥ 6.5% or on blood glucose lowering medication - Age of 18 - 75 years - Male and Female patients (females of child bearing potential must be using adequate contraceptive precautions) - Females of childbearing potential or within two years of the menopause must have a negative urine pregnancy test at screening visit |
| Exclusion criteria | - age over 75 years - Any other form of diabetes mellitus than type 2 diabetes mellitus - History of diabetic ketoacidosis or hyperosmolar nonketotic coma - Patients with more than one oral blood glucose lowering medication or on insulin therapy - Any medication with loop diuretics - Last measured HbA1c ≥ 10% (within last two weeks to screening visit or measured during the study) - Fasting blood glucose > 240mg/dl - Blood pressure levels ≥180/110 mmHg - Estimated glomerular filtration rate (eGFR < 60 ml/min/1.73m²) - Acute cardiovascular event (including myocardial infarction, unstable angina pectoris, percutaneous coronary intervention, heart failure, stroke, TIA. PRIND, intracerebral bleeding) <3 months prior to screening visit (visit 0) - Body mass index >40 kg/m² - Triglyceride levels >1000 mg/dl - HDL-cholesterol levels <25 mg/dl - Macroalbuminuria defined by urinary albumine-to-creatinine ratio > 300 mg/g - Known liver function test >3 times upper limit of normal - Pregnant or breast-feeding patients - Any patient currently receiving chronic (>30 consecutive days) treatment with an oral corticosteroid - Subjects with a history of any serious hypersensitivity reaction to Dapagliflozin or SGLT-2 inhibitor - Presence of significant renal, respiratory, hepatic, gastrointestinal, endocrine or metabolic, immunological, haematological or oncological, neurological and psychiatric diseases or dysfunction - Diabetic retinopathy - History of epilepsia or history of seizures - Patients suffering from cataract or glaucoma - History of drug medication abuse. - Patients being treated for severe auto immune disease e.g. lupus | - Any other form of diabetes mellitus than type 2 diabetes mellitus - Use of insulin, glitazone, gliptine or SGLT-2 inhibitor within the past 3 months - Patients with more than one oral blood glucose lowering medication - Any other oral antidiabetic drug that cannot be discontinued for the study period - HbA1c ≥ 10% - Fasting plasma glucose > 240 mg/dl - Any history of stroke, transient ischemic attack, instable angina pectoris, or myocardial infarction within the last 6 months prior to study inclusion - UACR ≥ 300 mg/g (early morning spot urine) - eGFR < 60 ml/min/1.73m² - Uncontrolled arterial hypertension (RR ≥ 180/110 mmHg) - Congestive heart failure (CHF) NYHA stage III and IV - Severe disorders of the gastrointestinal tract or other diseases which interfere the pharmacodynamics and pharmakinetics of study drugs - Significant laboratory abnormalities such as SGOT or SGPT levels more than 3 x above the upper limit of normal range - Drug or alcohol abusus - Pregnant or breast-feeding patients - Use of loop diuretics - History of repetitive urogenital infection per year - Body mass index > 40 kg/m² - Triglyceride levels > 1000 mg/dl - HDL-cholesterol levels < 25 mg/dl - Any patient currently receiving chronic (>30 consecutive days) treatment with an oral corticosteroid - History of epilepsia or history of seizures - Patients being treated for severe auto immune disease e.g. lupus - Participation in another clinical study within 30 days prior to visit 1 - Individuals at risk for poor protocol or medication compliance |

Flow chart of the study design and procedures

Empagliflozin 25 mg/day

Placebo

Placebo

Wash-out

Run-in

4 weeks

6 weeks

6 weeks

1 week

Wash-out

Dapagliflozin 10 mg/day

Empagliflozin 25 mg/day

Dapagliflozin 10 mg/day

Examination and fasting blood and 24‑h urine sample collection

Examination and fasting blood and 24‑h urine sample collection

Examination and fasting blood and 24‑h urine sample collection

Double-blinded phase

**Composition of Placebo**^6,7^

Placebo for dapagliflozin tablet:

Microcrystalline cellulose (E460i), Anhydrous lactose (or lactose; each 5 mg tablet contains ~25 mg lactose; each 10 mg tablet contains ~50 mg lactose), Crospovidone (E1202), Silicon dioxide (E551; colloidal silica), Magnesium stearate (E470b).

The film coating is made of the following substances: Polyvinyl alcohol (E1203), Titanium dioxide (E171), Macrogol/polyethylene glycol 3350 (E1521), Talc (E553b) and yellow iron oxide (E172)

Placebo for empagliflozin tablet:

Lactose monohydrate, Microcrystalline cellulose, Hydroxypropyl cellulose, Croscarmellose sodium, Colloidal anhydrous silica (or colloidal silicon dioxide), Magnesium stearate.

The film coating is made of the following substances: Hypromellose, Titanium dioxide (E171), Talc, Polyethylene glycol (macrogol 400), Yellow iron oxide (E172)

| Sample preparation: | Blank matrix plasma: 5-fold dialysed plasma  Blank matrix urine: Sigmatrix Urine Diluent (Sigma-Aldrich/Merck, Darmstadt, Germany) |
| --- | --- |
| Instrument: | HPLC-MS-MS  Agilent 1100 HPLC System, API 4000 QQQ-MS |
| Column: | EC 100/2 Nucleoshell HILIC column (2.7 µm, 150 x 2 mm)  equipped with a guard column (4 x 2 mm) |
| Chromatography: | Gradient \| Isocratically; Mobile Phase \| 4 g/L ammonium formate in acetonitrile-water (75:25) pH 3 (adjusted with formic acid); Flow \| 0.15 ml/min; Temperature \| 20 °C; Injection volume \| 10 µl; Run time \| 5 min; TMAO retention time \| 2.1 min |
| MS/MS parameters: | MRM transitions used for MRM (ESI positive mode): Q1: precursor ion, Q3: product-ion, DP: decoupling potential, CE: collision energy, CXP: collision cell exit potential \| \| \| \|; \| Q1 → Q3 \| DP \| CE \| CXP; TMAO (Quantifier) \| 76.1 → 59.1 \| 51 \| 17 \| 4; TMAO (Qualifier) \| 76.1 → 58.1 \| 51 \| 29 \| 2; TMAO-d9 (Internal standard) \| 85.0 → 66.0 \| 66 \| 29 \| 4 Cycle time for each MRM transition \| 300 ms; Ion source temperature \| 500 °C |

**Further information on LC-MS/MS method**^8^

**Intraindividual correlations of plasma TMAO values**

| **Table S1** Intraindividual correlations of plasma TMAO values at baseline | | | | | | |
| --- | --- | --- | --- | --- | --- | --- |
|  | eGFR | | | Fasting blood glucose | | |
|  | N | r | p-value | N | r | p-value |
| Total | 123 | -0.406 | 0.000 | 123 | 0.063 | 0.491 |
| Empagliflozin study | 69 | -0.343 | 0.004 | 69 | -0.087 | 0.475 |
| Dapagliflozin study | 54 | -0.463 | 0.000 | 54 | 0.262 | 0.055 |
| Correlations are shown as Spearman-Rho correlation coefficients (r); eGFR, estimated glomerular filtration rate | | | | | | |

| **Table S2** Intraindividual correlations of plasma TMAO values after treatment with an SGLT2-inhibitor | | | | | | |
| --- | --- | --- | --- | --- | --- | --- |
|  | eGFR | | | Fasting blood glucose | | |
|  | N | r | p-value | N | r | p-value |
| Total | 123 | -0.307 | 0.001 | 123 | 0.061 | 0.500 |
| Empagliflozin study | 69 | -0.325 | 0.006 | 69 | 0.084 | 0.495 |
| Dapagliflozin study | 54 | -0.290 | 0.033 | 54 | -0.003 | 0.984 |
| Correlations are shown as Spearman-Rho correlation coefficients (r); eGFR, estimated glomerular filtration rate | | | | | | |

| **Table S3** Intraindividual correlations of plasma TMAO values after treatment with placebo | | | | | | |
| --- | --- | --- | --- | --- | --- | --- |
|  | eGFR | | | Fasting blood glucose | | |
|  | N | r | p-value | N | r | p-value |
| Total | 123 | -0.256 | 0.004 | 123 | 0.136 | 0.134 |
| Empagliflozin study | 69 | -0.120 | 0.326 | 69 | 0.046 | 0.708 |
| Dapagliflozin study | 54 | -0.431 | 0.001 | 54 | 0.251 | 0.067 |
| Correlations are shown as Spearman-Rho correlation coefficients (r); eGFR, estimated glomerular filtration rate | | | | | | |

**Intraindividual correlations of renal clearance of TMAO**

| **Table S4** Intraindividual correlations of renal clearance of TMAO at baseline | | | | | | |
| --- | --- | --- | --- | --- | --- | --- |
|  | eGFR | | | Fasting blood glucose | | |
|  | N | r | p-value | N | r | p-value |
| Total | 123 | 0.284 | 0.001 | 123 | -0.195 | 0.030 |
| Empagliflozin study | 69 | 0.341 | 0.004 | 69 | -0.126 | 0.301 |
| Dapagliflozin study | 54 | 0.218 | 0.114 | 54 | -0.229 | 0.096 |
| Correlations are shown as Spearman-Rho correlation coefficients (r); eGFR, estimated glomerular filtration rate | | | | | | |

| **Table S5** Intraindividual correlations of renal clearance of TMAO after treatment with an SGLT2-inhibitor | | | | | | |
| --- | --- | --- | --- | --- | --- | --- |
|  | eGFR | | | Fasting blood glucose | | |
|  | N | r | p-value | N | r | p-value |
| Total | 123 | 0.327 | 0.000 | 123 | 0.064 | 0.480 |
| Empagliflozin study | 69 | 0.334 | 0.005 | 69 | 0.014 | 0.910 |
| Dapagliflozin study | 54 | 0.301 | 0.027 | 54 | 0.223 | 0.105 |
| Correlations are shown as Spearman-Rho correlation coefficients (r); eGFR, estimated glomerular filtration rate | | | | | | |

| **Table S6** Intraindividual correlations of renal clearance of TMAO after treatment with placebo | | | | | | |
| --- | --- | --- | --- | --- | --- | --- |
|  | eGFR | | | Fasting blood glucose | | |
|  | N | r | p-value | N | r | p-value |
| Total | 123 | 0.185 | 0.040 | 123 | 0.035 | 0.702 |
| Empagliflozin study | 69 | 0.215 | 0.075 | 69 | 0.061 | 0.617 |
| Dapagliflozin study | 54 | 0.117 | 0.400 | 54 | 0.006 | 0.965 |
| Correlations are shown as Spearman-Rho correlation coefficients (r); eGFR, estimated glomerular filtration rate | | | | | | |

**Intraindividual correlations of treatment related change after treatment with SGLT2-inhibitor in comparison to treatment with placebo**

| **Table S7** Intraindividual correlations of treatment-related change in plasma after treatment with an SGLT2-inhibitor in comparison to treatment with placebo | | | | | | |
| --- | --- | --- | --- | --- | --- | --- |
|  | eGFR | | | Fasting blood glucose | | |
|  | N | r | p-value | N | r | p-value |
| Total | 123 | -0.026 | 0.774 | 123 | 0.152 | 0.094 |
| Empagliflozin | 69 | 0.108 | 0.375 | 69 | 0.125 | 0.307 |
| Dapagliflozin | 54 | -0.183 | 0.186 | 54 | 0.181 | 0.191 |
| Correlations are shown as Spearman-Rho correlation coefficients (r); eGFR, estimated glomerular filtration rate | | | | | | |

| **Table S8** Intraindividual correlations of treatment-related change in renal clearance after treatment with an SGLT2-inhibitor in comparison to treatment with placebo | | | | | | |
| --- | --- | --- | --- | --- | --- | --- |
|  | eGFR | | | Fasting blood glucose | | |
|  | N | r | p-value | N | r | p-value |
| Total | 123 | 0.110 | 0.224 | 123 | -0.067 | 0.465 |
| Empagliflozin | 69 | 0.214 | 0.078 | 69 | -0.050 | 0.686 |
| Dapagliflozin | 54 | 0.042 | 0.763 | 54 | -0.079 | 0.570 |
| Correlations are shown as Spearman-Rho correlation coefficients (r); eGFR, estimated glomerular filtration rate | | | | | | |

**Impact of concomitant intake of antibiotics**

| **Table S9** Influence of antibiotics – comparison between totalparticipants and participants with no intake of an antibiotic | | | |
| --- | --- | --- | --- |
|  | **Total N=123** | **No antibiotic N=103** | **p-value** |
| TMAO plasma crossover difference [µmol/l] | 0.36 (-0.56 – 1.76) | 0.36 (-0.56 – 1.97) | 0.902 |
| TMAO Amount excreted in 24h urine crossover difference [µmol] | 24.3 (-184.6 – 196.7) | 41.7 (-166.2 – 229.1) | 0.696 |
| TMAO renal clearance crossover difference [ml/min] | -9.5 (-49.9 – 24.0) | -9.5 (-48.2 – 26.5) | 0.992 |
| Data presented as median (IQR). p-values calculated by Mann-Whitney-U-Test. | | | |

| **Table S10** Influence of antibiotics – comparison between participants with no intake of an antibiotic and participants with intake of an antibiotic | | | |
| --- | --- | --- | --- |
|  | **No antibiotic N=103** | **antibiotic N=20** | **p-value** |
| TMAO plasma crossover difference [µmol/l] | 0.36 (-0.56 – 1.97) | 0.24 (-0.42 – 1.55) | 0.681 |
| TMAO Amount excreted in 24h urine crossover difference [µmol] | 41.7 (-166.2 – 229.1) | -42.1 (-317.3 – 139.0) | 0.190 |
| TMAO renal clearance crossover difference [ml/min] | -9.5 (-48.2 – 26.5) | -3.7 (-56.2 – 21.4) | 0.973 |
| Data presented as median (IQR). p-values calculated by Mann-Whitney-U-Test. | | | |

**References:**

1.	Gessner A, Gemeinhardt A, Bosch A, et al. Effects of treatment with SGLT-2 inhibitors on arginine-related cardiovascular and renal biomarkers. *Cardiovasc Diabetol*. 2022;21(1):4. doi:10.1186/s12933-021-01436-x

2.	Ott C, Jumar A, Striepe K, et al. A randomised study of the impact of the SGLT2 inhibitor dapagliflozin on microvascular and macrovascular circulation. *Cardiovasc Diabetol*. 2017;16(1):26. doi:10.1186/s12933-017-0510-1

3.	Bosch A, Ott C, Jung S, et al. How does empagliflozin improve arterial stiffness in patients with type 2 diabetes mellitus? Sub analysis of a clinical trial. *Cardiovasc Diabetol*. 2019;18(1):44. doi:10.1186/s12933-019-0839-8

4.	Striepe K, Jumar A, Ott C, et al. Effects of the selective sodium-glucose cotransporter 2 inhibitor empagliflozin on vascular function and central hemodynamics in patients with type 2 diabetes mellitus. *Circulation*. 2017;136(12):1167-1169. doi:10.1161/CIRCULATIONAHA.117.029529

5.	Karg MV, Bosch A, Kannenkeril D, et al. SGLT-2-inhibition with dapagliflozin reduces tissue sodium content: a randomised controlled trial. *Cardiovasc Diabetol*. 2018;17:5. doi:10.1186/s12933-017-0654-z

6.	Forxiga: EPAR - product information. https://www.ema.europa.eu/en/medicines/human/EPAR/forxiga. Accessed August 13, 2025.

7.	Jardiance: EPAR - product information. https://www.ema.europa.eu/en/medicines/human/EPAR/jardiance. Accessed August 13, 2025.

8.	Gessner A, Di Giuseppe R, Koch M, Fromm MF, Lieb W, Maas R. Trimethylamine-N-oxide (TMAO) determined by LC-MS/MS: Distribution and correlates in the population-based PopGen cohort. *Clin Chem Lab Med CCLM*. 2020;58(5):733-740. doi:10.1515/cclm-2019-1146
